# Dietary Polyphenols and Their Effects on Cell Biochemistry and Pathophysiology 2013

**DOI:** 10.1155/2014/576363

**Published:** 2014-02-05

**Authors:** Tullia Maraldi, David Vauzour, Cristina Angeloni

**Affiliations:** ^1^Department of Surgical, Medical, Dental and Morphological Sciences with Interest in Transplant, Oncology and Regenerative Medicine, University of Modena and Reggio Emilia, 41124 Modena, Italy; ^2^Department of Nutrition, Norwich Medical School, University of East Anglia, Norwich NR4 7TJ, UK; ^3^Department for Life Quality Studies, Alma Mater Studiorum University of Bologna, 40126 Bologna, Italy

## 1. Introduction

Dietary polyphenols, along with other natural compounds occurring in fruits and vegetables, have been reported to exert beneficial effects in a multitude of disease states, including cancer, cardiovascular disease, and neurodegenerative disorders. Many of the biological actions of polyphenols have been attributed to their antioxidant properties; however, during the last years, a new realization of how nutritional antioxidants may function has been envisaged, and recent findings have suggested that they affect several cellular pathways exerting a pleiotropic effect.

This special issue analyzes and expands our knowledge on the new mechanisms of actions of polyphenols and other natural compounds with the aim to better understand what could be defined as “the network” of their different biological effects.

## 2. Role of Polyphenols in Redox Modulation and Inflammatory Processes

Although moderate physical exercise is considered an essential component of a healthy lifestyle that leads the organism to adapt itself to different stresses, strenuous exercise is however known to induce oxidative stress, inflammation, and muscle damage. The review of M. Malaguti et al. focused on polyphenols, present in the plant kingdom, that have been recently suggested to exert some positive effects on muscle damage and oxidative stress induced by vigorous physical exercise.

In the study of L. D'evoli et al., the nutraceutical effects of red chicory extracts were evaluated in various intestinal models. Compared to red chicory whole leaf extracts, the red part of leaf extracts had a significantly higher content of both total phenolics and anthocyanins, leading to an increase of antioxidant, cytoprotective, and antiproliferative activities. The study of I. Chkhikvishvili et al. analyzed the effects of summer savory extracts in counteracting H_2_O_2_-induced oxidative stress in human lymphoblastoid Jurkat T cells. In particular they demonstrated that summer savory extracts protective effect may be attributed to the direct radical-scavenging activity of rosmarinic acid and other phenolic compounds, as well as to indirect mechanisms such as the enhancement of antioxidant enzymes and the release of anti-inflammatory signaling molecules, such as IL-10.

C. Wall et al. demonstrated that luteolin and kaempferol decrease IL-1*β*-induced NF-*κ*B p65 DNA binding activity and nuclear c-Jun expression in human gestational tissues, suggesting a potential role of these compounds in counteracting infection/inflammation, commonly associated with preterm birth, initiating uterine contractions and rupture of fetal membranes.

Neutrophils are capable of releasing cytotoxic substances and inflammatory mediators, which, along with their delayed apoptosis, have a potential to maintain permanent inflammation.

V. Jancinova et al. investigated the ability of piceatannol, a naturally occurring stilbenoid, in reducing the toxic effect of neutrophils. This stilbenoid elevates the percentage of early apoptotic neutrophils and inhibits the activity of protein kinase C (PKC), the main regulatory enzyme in neutrophils, indicating that piceatannol may be useful as a complementary medicine in states associated with persisting neutrophil activation and with oxidative damage of tissues.

In the study of M.-J. Bak et al., the anti-inflammatory effect and underlying mechanisms of wild grape seeds procyanidins (WGP) were examined in RAW 264.7 cells. They demonstrated that WGP exerts potent anti-inflammatory activity through the inhibition of iNOS and COX-2 and by regulating NF*κ*B and p38 MAPK pathways. R. Nosàl et al. showed that resveratrol represents an effective naturally occurring substance with potent pharmacological effect on oxidative burst of human neutrophils and nitric oxide production by macrophages.

K. Drábiková et al., with the aim to specify the site of action of the synthetic coumarin derivatives 7-hydroxy-3-(4′-hydroxyphenyl) coumarin (HHC) and 7-hydroxy-3-(4′-hydroxyphenyl) dihydrocoumarin (HHDC), evaluated their effects on extra- and intracellular ROS formation in phorbol-myristate-13-acetate (PMA) stimulated human neutrophils. Without affecting cytotoxicity, both tested coumarins were effective inhibitors/scavengers of ROS produced by neutrophils on extracellular level. HHC markedly diminished oxidant production and also decreased both PKC activity and phosphorylation of PKC*α*, *β*II intracellularly.

Oxidative stress plays a major role in the pathophysiology of chronic inflammatory disease and it has also been linked to accelerated telomere shortening. Research has shown that poly(ADP-ribose) polymerase-1 (PARP-1) and subtelomeric methylation play a role in telomere stability. D. M. P. H. J. Boesten et al., evaluating the effect of chronic PARP-1 inhibition (by fisetin and minocycline) in human fibroblasts exposed to chronic oxidative stress, demonstrated that PARP-1 plays a role in accelerated aging in chronic inflammatory diseases due to its role as coactivator of NF-*κ*B and AP-1.

## 3. Role of Polyphenols in Molecular Signaling

The NADPH oxidase (NOX) physiological functions concern host defense, cellular signaling, regulation of gene expression, and cell differentiation. On the other hand, increased NOX activity contributes to a wide range of pathological processes, including cardiovascular diseases, neurodegeneration, organ failure, and cancer. The review by I. Maraldi summarizes the current state of knowledge of the role of NOX enzymes in physiology and pathology and provides an overview of the currently available NOX inhibitors derived from natural extracts such as polyphenols.

In several species, the polyphenol resveratrol activates sirtuins (SIRT). SIRT1, a NAD^+^-dependent deacetylase, was identified as one of the molecules through which calorie restriction extends the lifespan or delays age-related diseases, regulating adaptations to cellular stress, through the deacetylation of target proteins. F. Pifferi et al. showed a reduction in locomotor activity onset and changes in body temperature rhythm in resveratrol-supplemented aged animals suggesting an improved synchronisation on the light-dark cycle through SIRT-regulation of energy balance and biological clock processes.

Moreover the review by M. Kitada and D. Koya focused on resveratrol and its beneficial effects on renal diseases. They evidenced that resveratrol can exert protective effects against both acute and chronic kidney injuries through its antioxidant effects and ability to activate SIRT1. The regulation of SIRT1 was proposed also by K. Pallauf et al. suggesting that the so-called “MediterrAsian” diet combining sirtuin-activating foods of the Asian as well as Mediterranean diet may be a promising dietary strategy in preventing chronic diseases, thereby ensuring health and healthy ageing.

## 4. Role of Polyphenols in Stem Cell Proliferation and Differentiation

Human stem cells with multilineage differentiation potential are studied for cell therapy, but *in vitro* expansion leads to senescence affecting differentiation and proliferative capacities. M. Guida et al. demonstrated that with a decrease of Nox4 activity in stem cells, obtained with plumbagin, a decline of nuclear ROS production and of DNA damage occurs. They suggest that nuclear Nox4 regulation may have important pathophysiologic effects in stem cell proliferation through modulation of nuclear signaling and DNA damage.

## 5. Role of Polyphenols in Metabolism Regulation 

Extracts from *Stevia rebaudiana* Bertoni are largely used as a noncaloric, high-potency biosweetener alternative to sugar, due to the growing incidence of type 2 diabetes mellitus, obesity, and metabolic disorders worldwide. B. Rizzo et al. showed that these extracts are able to enhance glucose uptake in different cell lines and were as efficient as insulin at modulating PI3K/Akt pathway.

Nonalcoholic fatty liver disease, defined by excessive lipid accumulation in the liver, is the hepatic manifestation of insulin resistance and the metabolic syndrome. L. Valenti et al. summarize the evidence evaluating the mechanisms of action of anthocyanins on hepatic lipid metabolism in different experimental settings including cells, animals, and human trials. Data shown in the paper by E. Giaretta et al. demonstrate that resveratrol supplementation in various phases of *in vitro* oocyte maturation and vitrification/warming procedure can modulate the apoptotic process, improving the resistance of porcine oocytes to cryopreservation-induced damage.

## 6. Potential Effect of Polyphenols in Cancer

M. Del Carmen García-Rodríguez et al. investigated the modulating effects of green tea polyphenols on genotoxic damage and apoptotic activity induced by hexavalent chromium (Cr (VI)) in mice. Their findings supported the protective effects of green tea polyphenols by inducing apoptosis that could contribute to elimination of the DNA damaged cells induced by Cr (VI).

Y. Ke et al. evaluated the potential protective effects of Fructus rhodomyrti (FR) extracts against oxidative DNA damage. Their findings suggested that FR might act as a chemopreventive agent with antioxidant properties offering effective protection against oxidative DNA damage in a concentration-dependent manner *in vitro* and *in vivo*. Quercetin is a dietary flavonoid with known antitumor effects against several types of cancers by promoting apoptotic cell death and inducing cell cycle arrest. H. Kim et al. showed that quercetin is significantly effective in inhibiting cell proliferation through mitochondrial apoptosis pathway but also inducing autophagy.

## 7. Potential Effect of Polyphenols in Cardiovascular Diseases

Epigallocatechin gallate (EGCG) is known to exhibit antioxidant, antiproliferative, and antithrombogenic effects and reduce the risk of cardiovascular diseases. Key events in the development of cardiovascular disease are hypertrophy and hyperplasia according to vascular smooth muscle cell proliferation. M. H. Lee et al. suggest that EGCG can inhibit PDGF-BB stimulated mitogenesis by indirectly and directly interrupting PDGF-BB pathway. Therefore, EGCG may be used for treatment and prevention of cardiovascular disease through blocking of PDGF signaling.

## 8. Potential Effect of Polyphenols in Neurodegenerative Diseases

The role of oxidative stress in age-related dementia and in stroke pathophysiology is crucial; therefore the use of antioxidant plant derivatives is under investigation. In the studies by C. Sutalangka et al. and W. Kirisattayakul et al. the neuroprotective effect of *Moringa oleifera*, a plant possessing potent antioxidant activity, was assessed in animal models of age-related dementia and focal stroke. Their data showed that *M. oleifera* leaves extract is neuroprotectant and cognitive enhancer partly via the decreased oxidative stress and the enhanced cholinergic function.

Moreover they showed that all extract doses decreased infarction volume in both cortex and subcortex. The protective effect of low extract doses in all areas occurs mainly via the decreased oxidative stress. The protective effect of the high dose extract in striatum and hippocampus occurs via the same mechanism, whereas other mechanisms might play a crucial role in cortex.

1-Methyl-4-phenyl-1,2,3,6-tetrahydropyridine (MPTP) is an environmental toxin which selectively induces oxidative damage and mitochondrial and proteasomal dysfunctions to dopaminergic neurons in the substantia nigra leading to Parkinsonian syndrome in animal models and humans. N. Braidy et al. investigated the therapeutic effect of different varieties of pomegranate juice extracts (PJE), Helow, Malasi, Qusum, and Hamadh, against MPTP-induced neurotoxicity in primary human neurons. Helow and Malasi show the best effect in attenuating the observed changes in redox function thus ameliorating MPTP-induced neurotoxicity.

Rotenone, a widely used pesticide that inhibits mitochondrial complex I, has been used to investigate the pathobiology of Parkinson's disease. Studies have shown that the neurotoxicity of rotenone may be related to its ability to generate ROS, leading to neuronal apoptosis. The study of K. Tamilselvam et al. was carried out to investigate the neuroprotective effects of hesperidin, a citrus fruit flavanol, against rotenone-induced apoptosis in human neuroblastoma cells. The data suggested that hesperidin exerts its neuroprotective effect against rotenone due to its antioxidant, maintenance of mitochondrial function, and antiapoptotic properties.

*Tullia Maraldi* *Tullia Maraldi* *David Vauzour* *David Vauzour* *Cristina Angeloni* *Cristina Angeloni*

